# Supplementary material for: Insights into long-acting reversible contraceptive practices in Sub-Saharan Africa: A machine learning perspective
Source: PLoS One. 2026 Jan 20;21(1):e0330960. doi: 10.1371/journal.pone.0330960 (PMC12818626; doi:10.1371/journal.pone.0330960)
Supplement: Attachment — Submitted filename: review.pdf [file pone.0330960.s001.pdf]

Review Report

Manuscript ID: PONE-D-25-24936
Title: Insights into Long-Acting Reversible Contraceptive Practices in Sub-Saharan Africa: A Machine Learning Perspective
Reviewer Recommendation: Minor Revision

1 Strengths

- **Timely and Policy-Relevant Topic:** The low uptake of LARCs in SSA is a significant public health concern. The focus aligns with SDGs and reproductive health equity.
- **Use of Modern ML Techniques:** The application of eight ML models with tuning and SHAP-based interpretation represents a novel and appropriate methodological advancement in the field.
- **Open Science Compliance:** The manuscript provides open access to data and code, aligning with PLOS standards for reproducibility.
- **Structured and Transparent Workflow:** The authors follow a well-documented process for preprocessing, modeling, evaluation, and interpretation.

2 Major Suggestions for Improvement

Model Performance and Class Imbalance

While SMOTE was applied, the minority class (LARC users, $\sim$3.3%) remains difficult to model. Precision and recall metrics, especially before tuning, were low.
Suggestion: Consider evaluating ensemble undersampling (e.g., SMOTEENN), focal loss, or adjusting classification thresholds to optimize the precision-recall balance. Additionally, reporting confidence intervals (e.g., via DeLong's test) for AUC-ROC would enhance robustness claims.

Validation Strategy

The current strategy uses stratified cross-validation, which is valid, but may overestimate generalizability in cross-national datasets.
Suggestion: Implement a leave-one-country-out validation strategy or report per-country performance to highlight model robustness and geographic heterogeneity.

Feature Engineering

Several features had high missingness but were retained after imputation without strong justification. Also, the SHAP analysis revealed low importance for some variables (e.g., religion).

Suggestion: Conduct feature selection using SHAP or mutual information to reduce noise. Also, consider deriving interaction terms (e.g., age × household size, education × media exposure) to improve non-linear predictive power.

Interpretation of AUC

The reported AUC-ROC of 0.72 is modest. While sufficient, claims of "strong predictive capability" may be overstated.

Suggestion: Temper conclusions and emphasize precision-recall tradeoffs more prominently in both the results and conclusion.

Opportunity for Broader Contextualization

The study could benefit from incorporating contextual features (e.g., health facility density, national FP program intensity) to explain cross-country variation.

Suggestion: Acknowledge this in the limitations and suggest it as a direction for future research.

3 Minor Comments

- Several typographic errors and verbose sections could be edited for clarity (e.g., overuse of "data-driven," "actionable insights," etc.).
- Figures (especially ROC, PR curves) could benefit from improved captions explaining key takeaways.
- SHAP force plots and examples should be more explicitly discussed to help non-technical readers understand implications.

4 Ethical and Data Compliance

- Ethical review and consent are well-explained and meet standards for secondary data analysis.
- Data and code are publicly available via the PMA and GitHub, consistent with open science principles.

5 Conclusion & Recommendation

This is a well-structured, relevant, and technically sound manuscript. While the performance of the models is modest, the study contributes meaningfully to public health analytics in SSA and demonstrates a reproducible ML framework for policy insight.

Recommendation: Minor Revision

Required Actions Before Acceptance:

- Clarify how class imbalance was managed and consider additional methods (SMOTE variants or class weights).
- Add model confidence intervals (e.g., AUC error bars or statistical significance).
- Expand discussion of limitations, especially around generalizability and feature limitations.
- Reframe overstatements of model accuracy or predictive power.
