# Supplementary material for: Insights into long-acting reversible contraceptive practices in Sub-Saharan Africa: A machine learning perspective
Source: PLoS One. 2026 Jan 20;21(1):e0330960. doi: 10.1371/journal.pone.0330960 (PMC12818626; doi:10.1371/journal.pone.0330960)
Supplement: Attachment — Submitted filename: Response to reviewres.docx [file pone.0330960.s003.docx]

July 15, 2025

To: Editors of PLOS One

Response to reviewers’ comments

We want to thank you for inviting us to resubmit our manuscript entitled **“Insights into Long-Acting Reversible Contraceptive Practices in Sub-Saharan Africa: A Machine Learning Perspective”**. We are grateful to the reviewers for their time and effort in providing us with their valuable comments on our manuscript. We appreciate the comments the reviewers have provided to help improve this manuscript. We have noted all the comments and tried to address them in the revised manuscript accordingly. Moreover, we have thoroughly revised the grammatical correctness throughout this manuscript and incorporated all the suggestions provided by the reviewers. Point-by-point responses to the reviewer’s concerns are indicated below.

**Reviewer Comments:**

**Major Suggestions for Improvement**

**Model Performance and Class Imbalance**

While SMOTE was applied, the minority class (LARC users, ∼3.3%) remains difficult to

model. Precision and recall metrics, especially before tuning, were low.

Suggestion: Consider evaluating ensemble undersampling (e.g., SMOTEENN), focal

loss, or adjusting classification thresholds to optimize the precision-recall balance. Ad

ditionally, reporting confidence intervals (e.g., via DeLong’s test) for AUC-ROC would

enhance robustness claims.

Response: Thank you for your insightful suggestions. In response, we have implemented the **SMOTEENN ensemble resampling technique** to better address the class imbalance and improve model performance for the minority class (LARC users). This approach helped enhance both precision and recall metrics across several classifiers.

We appreciate your recommendation and believe this adjustment has contributed meaningfully to the robustness of our modeling strategy.

**Validation Strategy**

The current strategy uses stratified cross-validation, which is valid, but may overestimate

generalizability in cross-national datasets.

Suggestion: Implement a leave-one-country-out validation strategy or report per country

performance to highlight model robustness and geographic heterogeneity.

Response: Thank you for the valuable suggestion regarding the validation strategy. We agree that in many cases, a leave-one-country-out (LOCO) approach or reporting per-country performance can provide useful insights, especially when the goal is to assess generalizability across heterogeneous contexts. However, the primary aim of this study is to evaluate model performance at a regional level across Sub-Saharan Africa, rather than making country-specific inferences. The data were pooled intentionally to reflect shared regional dynamics, with a harmonized feature set across countries to capture overarching patterns rather than country-level nuances.

As such, stratified cross-validation was selected to preserve the class balance and ensure robust learning across the collective dataset. While we acknowledge that this may not fully capture potential country-level heterogeneity, we believe it aligns with the scope and goals of this study. Nevertheless, we have added a discussion of this point in the limitations section to acknowledge the trade-off and to encourage future research to explore country-specific models where appropriate.

**Feature Engineering**

Several features had high missingness but were retained after imputation without strong

justification. Also, the SHAP analysis revealed low importance for some variables (e.g.,

religion).

Suggestion: Conduct feature selection using SHAP or mutual information to reduce

noise. Also, consider deriving interaction terms (e.g., age × household size, education ×

media exposure) to improve non-linear predictive power.

Response: Thank you for your thoughtful feedback and valuable suggestions.

In response to your concerns regarding high missingness and feature importance:

- We conducted **feature selection using SHAP values**, focusing on the top 15 most informative features. Variables with consistently low SHAP importance (e.g., *religion*) were excluded from the final model.
- To address missingness, we applied **KNN imputation** and ensured that features with excessive missingness and no predictive value were removed.
- Additionally, we incorporated **interaction terms**, which are recommended by you (age *× household size* and *education × media exposure),* to capture potential non-linear relationships, as recommended.
- As a result, our model performs better than before

**Interpretation of AUC**

The reported AUC-ROC of 0.72 is modest. While sufficient, claims of “strong predictive

capability” may be overstated.

Suggestion: Temper conclusions and emphasize precision-recall tradeoffs more promi

nently in both the results and conclusion.

Response: Thank you for your thoughtful feedback. At the time of the initial submission, the model's AUC-ROC was indeed moderate (0.72), and we agree that the phrasing regarding "strong predictive capability" may have been optimistic. However, after incorporating additional improvements and refinements, the model now demonstrates significantly stronger performance, with metrics that better justify the original wording. We have also revised the manuscript to more clearly highlight the precision-recall tradeoffs, especially in the updated results and conclusion sections, to ensure a balanced interpretation of the model’s predictive strengths.

**Opportunity for Broader Contextualization**

The study could benefit from incorporating contextual features (e.g., health facility den

sity, national FP program intensity) to explain cross-country variation.

Suggestion: Acknowledge this in the limitations and suggest it as a direction for future

research.

Response: Thank you for this insightful recommendation. We agree that incorporating contextual features such as health facility density, national family planning (FP) program intensity, or policy-level indicators could provide a richer understanding of cross-country variation and improve the model's explanatory power. However, such contextual variables were beyond the scope of the current analysis, which focused primarily on individual- and household-level predictors available consistently across countries.

We have now acknowledged this limitation in the revised manuscript and suggested the integration of contextual and programmatic variables as an important direction for future research to enhance model interpretability and policy relevance.

**Minor Comments**

• Several typographic errors and verbose sections could be edited for clarity (e.g.,

overuse of “data-driven,” “actionable insights,” etc.).

Response: Thank you for pointing this out. We have carefully reviewed the manuscript to correct typographic errors and improve clarity. Redundant phrases such as repeated use of “data-driven” and “actionable insights” have been revised or removed to enhance readability and precision. We appreciate your attention to detail, which has helped improve the overall quality of the writing.

• Figures (especially ROC, PR curves) could benefit from improved captions explain

ing key takeaways.

Response: Thank you for this helpful suggestion. We have revised the figure captions, particularly for the ROC and Precision-Recall curves, to provide clearer explanations of their relevance and key takeaways. The updated captions now highlight the model’s performance characteristics, such as trade-offs between sensitivity and precision, and contextualize their implications for decision-making. We believe these enhancements improve the interpretability and value of the visualizations.

• SHAP force plots and examples should be more explicitly discussed to help non

technical readers understand implications

Response: Thank you for this important observation. We agree that a clearer interpretation of SHAP force plots is essential for broader accessibility. In the revised manuscript, we have expanded the discussion around the SHAP visualizations to better explain what the force plots represent, how specific features influence individual predictions, and what these implications mean in practical terms. These additions are aimed at making the interpretability results more accessible to non-technical readers, including policymakers and public health practitioners.
