# Supplementary material for: Insights into long-acting reversible contraceptive practices in Sub-Saharan Africa: A machine learning perspective
Source: PLoS One. 2026 Jan 20;21(1):e0330960. doi: 10.1371/journal.pone.0330960 (PMC12818626; doi:10.1371/journal.pone.0330960)
Supplement: Attachment — Submitted filename: response to reviewers.docx [file pone.0330960.s004.docx]

Point-by-Point Response

**Additional Editor Comments:**

**Major Comments**
1. Implausible Model Performance – Possible Data Leakage
Reporting an AUC-ROC of 1.00 for Random Forest and 0.99 for other models is highly implausible for behavioral survey data with 3.3% positive class prevalence.
Such performance almost certainly indicates data leakage or resampling before train/test split.
Please clarify whether SMOTEENN resampling was applied before or within cross-validation folds.
An external validation or leave-one-country-out (LOCO) approach is essential to confirm true predictive ability.

Response: We sincerely thank the Editor for raising this important point. In the revised analysis, **SMOTEENN resampling is now applied strictly to the training data only after the train–test split**, ensuring that no information from the test set is used during model training. This was implemented using an imbalanced-learn pipeline, which guarantees that SMOTEENN is executed within each training fold of the cross-validation process. The corresponding Python code demonstrating this workflow is available in our GitHub repository and can be verified for reproducibility.

Regarding the suggestion for an **external validation or Leave-One-Country-Out (LOCO) approach**, we addressed this comment in our previous response. We agree that such approaches are valuable when the aim is to assess generalizability across heterogeneous contexts. However, as clarified earlier, the **primary goal of this study** is to evaluate model performance at a **regional level across Sub-Saharan Africa**, rather than to derive country-specific conclusions. The data were intentionally pooled and harmonized to capture shared regional dynamics rather than national differences.

Therefore, we employed **stratified cross-validation** to preserve class balance and ensure robust learning across the integrated dataset. We acknowledge that this design may not fully capture country-level variability; accordingly, a discussion of this trade-off and a recommendation for future country-level validation are now included in the Limitations section of the revised manuscript.

2. Validation and Generalizability
Stratified cross-validation across a pooled dataset ignores geographic heterogeneity.
Given that the dataset covers nine diverse countries, performance may be inflated by within-country homogeneity.
Please add either:
LOCO validation results, or
Per-country AUC, precision, and recall to assess model transferability.
Without this, the model’s regional claims are unsupported.

Response: We appreciate the Editor’s thoughtful feedback on model validation and generalizability. As noted in our previous response, we fully recognize the value of a Leave-One-Country-Out (LOCO) approach or per-country performance reporting when the objective is to assess **cross-country transferability**. However, the **goal of this study** is to evaluate model performance at a **regional level** across Sub-Saharan Africa, not to build or compare country-specific prediction models.

The dataset was intentionally **pooled and harmonized across nine countries** to capture **shared behavioral and demographic dynamics** influencing long-acting reversible contraceptive (LARC) use at a regional scale. Accordingly, we adopted **stratified cross-validation**, which maintains class balance while ensuring that each fold includes representative samples from all countries. This approach was selected to reflect the integrated nature of the dataset and the study’s focus on regional rather than national inference.

We acknowledge that this design may not fully address potential **geographic heterogeneity** or **country-specific effects**. To reflect this important consideration, we have expanded the Limitations section of the revised manuscript to explicitly note this trade-off and to encourage future work to explore LOCO or country-level validation frameworks for assessing national model transferability.

3. Feature Selection and Conceptual Interpretation
Religion, marital status, and urban/rural residence were excluded for “low SHAP importance,” yet decades of evidence show these are key determinants of contraceptive behavior in SSA.
Their exclusion likely reflects model bias or mis-specification rather than true irrelevance.
The authors should:
Reintroduce these variables in sensitivity analyses, or
Discuss the implications of excluding sociocultural predictors in the limitations.

Response: We thank the Editor for this critical insight. We fully agree that the exclusion of established sociocultural predictors like religion, marital status, and urban/rural residence is a significant limitation, not of the variables' real-world importance, but of our model's ability to capture their nuanced, context-specific effects.

In direct response to your suggestion to discuss the implications, we have added the following paragraph to the **"Limitations of the Study"** section:

"Furthermore, the exclusion of key sociocultural variables (e.g., religion, marital status) due to low SHAP importance represents a critical conceptual limitation. While this was a data-driven decision for model parsimony, it contradicts extensive evidence of their role in contraceptive decision-making in SSA. This discrepancy likely stems from our region-level analysis, which may dilute country-specific cultural nuances, and suggests that these factors may act as distal contextual drivers rather than direct individual-level predictors in a pooled model. Their omission underscores a potential gap in the model's translational relevance for designing deeply contextualized interventions."

We believe this addition strengthens the manuscript by providing a more critical and nuanced interpretation of our feature selection process. Thank you for prompting this important clarification.

4. Overstatement of Findings
The discussion repeatedly claims “unprecedented predictive capability” and “perfect discrimination.”
Such language is misleading and inconsistent with the modest performance (AUC ≈ 0.72) before tuning.
Please temper these statements and contextualize results within the limits of secondary, cross-sectional data.

Response: We thank the Editor for this crucial feedback on the tone of our findings. We agree that terms like "unprecedented predictive capability" and "perfect discrimination" were overly strong, especially in the context of the model's baseline performance.

In response, we have thoroughly revised the manuscript to temper these statements and provide a more balanced and accurate interpretation of the results. The changes emphasize that the high performance was achieved after extensive optimization and should be contextualized within the limitations of the data.

Key revisions include:

- In the Abstract: The phrase "unprecedented predictive capability" has been replaced with: "Optimized ML models demonstrate a high predictive capability for LARC adoption drivers..."
- In the Results and Discussion: We have removed the word "perfect" and reframed the description of the Random Forest model's performance (AUC-ROC: 1.00) to highlight that this was the result of rigorous tuning and balancing, and we have added caveats. For example:
  - Previous: "Random Forest achieved perfect discrimination..."
- Revised: "After hyperparameter tuning and class balancing, Random Forest achieved excellent discriminative performance (AUC-ROC: 1.00). In the Conclusion: We have reframed the conclusion to be more modest, focusing on the "potential" of ML and the "utility" of the approach, rather than making definitive claims of perfection.

We believe these revisions present our findings more accurately and responsibly, aligning the language with the methodological context and inherent limitations of cross-sectional, secondary data. Thank you for this important suggestion.

5. Methodological Transparency
The manuscript lacks clarity on several key analytical steps:
Which variables had >20% missingness and were excluded?
Were imputations performed separately per country or on pooled data?
How was the k parameter in k-NN imputation selected?
These details are critical for reproducibility.

Response: We thank the Editor for this important request for clarity. We have revised the **"Data Preprocessing"** section to explicitly list the variables and their missingness percentages to ensure full reproducibility. The text now clearly states:

- **The variable 'facility_fp_discussion' (21.33% missing) was the only variable with >20% missingness and was removed.**
- Variables with <10% missingness that were imputed using k-NN (k=5) on the pooled dataset are now explicitly listed with their percentages: "wealth_quintile" (14.31%), "ur" (14.31%), "marital_status" (13.41%), "fp_side_effects" (12.47%), "religion" (7.52%), "age_at_first_use" (4.94%), and others as detailed in the manuscript.
- The selection of **k=5** for k-NN imputation is now justified as a standard, robust value that balances local accuracy and noise reduction.

These details provide the necessary transparency for other researchers to replicate our analytical workflow.

6. Interpretation of SHAP Values
SHAP importance scores are reported, but their direction and implications are not clearly discussed.
Please include partial dependence or SHAP summary plots showing how key variables (e.g., age, household size) influence LARC use probability.
Discuss SHAP limitations in socio behavioral contexts — interpretability is not causality.

Response: We thank the Editor for this insightful suggestion to deepen our interpretation of the SHAP results. We agree that understanding the direction of feature effects and the limitations of SHAP is crucial for meaningful public health insights.

In response, we have made the following additions to the manuscript:

1. **In the "Results" section (under "Feature Importance"):** We have replaced the existing description with a new paragraph that explicitly states the direction of the effect for the top predictors and direction of all key features.
2. **In the "Limitation” section:** We have added a new paragraph discussing the limitations of SHAP values, clearly stating that they indicate association within the model, not causation in the real world.

7. Policy Implications
The policy recommendations (e.g., “mobile clinics for young women in large households”) are reasonable but not strongly derived from the model.
Please link each recommendation explicitly to specific predictor findings or patterns in the data.

Response: We thank the editor for this valuable suggestion. We agree that explicitly linking the policy recommendations to the specific model findings strengthens their justification.In response, we have revised the relevant sections in the **Abstract** and **Conclusion** to directly connect each recommendation to its underlying data-driven insight:

**Minor Comments**

Language and Style: The text is overly verbose, with frequent repetition of “data-driven” and “actionable insights.” Simplify and streamline technical descriptions.

Response: We thank the editor for this valuable feedback on improving the clarity and conciseness of our manuscript.

In response, we have conducted a thorough revision of the entire text to eliminate verbose language and repetitive phrases. Specifically, we have:

- Systematically removed overused terms such as "data-driven" and "actionable insights."
- Simplified complex and lengthy sentences for better readability.
- Replaced jargon with more direct and accessible language where possible.

We believe these edits have significantly improved the flow and precision of the manuscript without compromising the technical and scholarly content.

Figures and Captions: Figures (e.g., ROC and PR curves) require clearer captions explaining key takeaways and which models are being compared.

Response: We thank the editor for this important suggestion to improve the clarity of our figures. In response, we have revised all figure captions, particularly for the ROC and Precision-Recall (PR) curves to be more informative.

Tables: Table 7 (showing perfect performance) contradicts earlier Table 6 (AUC = 0.72). Please reconcile or explain this discrepancy.

Response: We thank the Editor for this astute observation, which allows us to clarify the sequential nature of our modeling pipeline. The discrepancy between **Table 6 (AUC ≈ 0.72)** and **Table 7 (AUC ≈ 1.00)** is not an error but a direct result of applying a critical additional preprocessing step.

- **Table 6** presents the results after hyperparameter tuning but **before** the application of the advanced class-balancing technique, SMOTEENN.
- **Table 7** presents the final results after applying **both hyperparameter tuning AND the SMOTEENN resampling** technique to the training data.

Statistical Reporting: Include 95% confidence intervals for AUC-ROC using DeLong’s test or bootstrapping. Consider including PR-AUC, which is more appropriate for imbalanced data.

Response: We thank the editor for this suggestion. We have now added 95% confidence intervals for AUC-ROC using DeLong's test and placed greater emphasis on Precision-Recall AUC in our evaluation, as these metrics are more appropriate for our imbalanced data. These enhancements strengthen the statistical rigor of our analysis.

Ethical and Data Section: Ethical compliance is satisfactory, but the description of data harmonization across countries could be expanded for clarity.

Response: We thank the reviewer for this valuable suggestion. In response, we have expanded the description of data harmonization in the **Methods** section. We now explicitly detail the process of pooling and standardizing variables across the nine countries, including the alignment of categorical responses, handling of country-specific codes, and the application of consistent preprocessing rules to ensure comparability. This clarification reinforces the validity of our cross-national analysis.

Reviewer #2: The manuscript presents an ambitious application of machine-learning (ML) methods to predict long-acting reversible contraceptive (LARC) use across Sub-Saharan Africa using PMA survey data. In its current form, it reads as a technically dense modeling exercise rather than a rigorously validated, policy-relevant study.

1. Reporting AUC-ROC = 1.00 with F1 = 0.98 for Random Forest and near-perfect metrics for other ensembles is statistically implausible for noisy, cross-national survey data with a 3.3 % minority class. The results suggest severe data leakage or overfitting (e.g., imputation applied before train-test split, SMOTE/SMOTEENN applied to the entire dataset, or lack of country-level separation). A re-run with strictly nested cross-validation and leave-one-country-out testing is essential. Confidence intervals for AUC and F1 should be reported.

Response: We thank the reviewer for their critical observation. In our revised analysis, SMOTEENN resampling and all preprocessing steps were applied strictly within the training folds to prevent data leakage. While the high performance may reflect overfitting to the resampled training distribution, our nested cross-validation and stratified design aimed to preserve regional representativeness. We acknowledge that perfect metrics are uncommon in survey-based studies and have tempered our conclusions accordingly. Confidence intervals for AUC and F1 are now reported, and we explicitly recommend external validation in future work.

2. Given the extreme class imbalance (3.3 % positive), AUC and accuracy are insufficient. Report precision–recall AUC, Matthews correlation coefficient, and calibration plots.

Response: We thank the reviewer for this suggestion. In the revised manuscript, we have expanded our evaluation beyond AUC and accuracy. We now report F1-score, precision, recall, and their trade-offs, along with Precision-Recall AUC. For the best-performing model, we also provide 95% confidence intervals for AUC-ROC using DeLong’s test. These additions offer a more nuanced view of model performance, particularly given the class imbalance.
3. Discuss clinical/operational trade-offs (false-positive vs. false-negative cost) in the context of limited health resources.

Response: Thank you for this valuable suggestion. We have added a discussion of the clinical and operational trade-offs between false positives and false negatives in the Discussion section, contextualizing model performance within the realities of limited public health resources.
4. The SHAP interpretation remains superficial. The authors must demonstrate how household size or age at first contraceptive use can be operationalized in interventions.

Response: We thank the reviewer for this insightful suggestion. To address it, we have added a new paragraph in the Discussion section that explicitly translates the top SHAP-identified predictors into actionable, operational strategies for public health programs. This clarifies how variables like household size and age at first use can directly inform the design of targeted interventions.
5. Present case-level SHAP examples tied to real-world decision-making (e.g., outreach prioritization) instead of generic force plots.

Response: We thank the reviewer for this excellent suggestion to enhance the practical interpretation of our SHAP results. In response, we have replaced the generic force plot discussion with concrete, contrasting case-level examples 
6. Claims such as “unprecedented predictive capability” and “ideal for deployment” are not supported.

Response: We thank the reviewer for this feedback. We have revised the manuscript to temper overstated claims, replacing phrases like “unprecedented predictive capability” and “ideal for deployment” with more measured language such as “strong performance” and “potential utility in resource-constrained settings.” These changes appear in the Abstract, Results, and Discussion sections.

7. The manuscript is verbose, with repetitive phrasing (“data-driven,” “actionable insights,” “transformative potential”). A professional language edit is recommended.

Response: We thank the editor for this valuable feedback on improving the clarity and conciseness of our manuscript.

In response, we have conducted a thorough revision of the entire text to eliminate verbose language and repetitive phrases. Specifically, we have:

- Systematically removed overused terms such as "data-driven" and "actionable insights."
- Simplified complex and lengthy sentences for better readability.
- Replaced jargon with more direct and accessible language where possible.

We believe these edits have significantly improved the flow and precision of the manuscript without compromising the technical and scholarly content.

8. Acronyms (MLP, SHAP, SMOTEENN) should be defined once and used consistently.

Response: Thank you for this helpful note. We have ensured that all acronyms (MLP, SHAP, SMOTEENN) are clearly defined upon first use in the manuscript and used consistently thereafter. A dedicated "Abbreviations and Acronyms" section has also been included for reference.
